# Optimized 2-methoxyestradiol invasomes fortified with apamin: a promising approach for suppression of A549 lung cancer cells

**DOI:** 10.1080/10717544.2022.2072412

**Published:** 2022-05-25

**Authors:** Zuhier A. Awan, Shareefa A. AlGhamdi, Nabil A. Alhakamy, Solomon Z. Okbazghi, Mohamed A. Alfaleh, Shaimaa M. Badr-Eldin, Hibah M. Aldawsari, Mohammed A. S. Abourehab, Hani Z. Asfour, Shadi A. Zakai, Mohammad W. Alrabia, Aya A. Negm, Mohamed A. El-Moselhy, Sara S. Sharkawi, Waleed Y. Rizg

**Affiliations:** a Department of Clinical Biochemistry, Faculty of Medicine, King Abdulaziz University, Jeddah, Saudi Arabia; b Department of Biochemistry, Faculty of Science, King Abdulaziz University, Jeddah, Saudi Arabia; c Department of Pharmaceutics, Faculty of Pharmacy, King Abdulaziz University, Jeddah, Saudi Arabia; d Center of Excellence for Drug Research and Pharmaceutical Industries, King Abdulaziz University, Jeddah, Saudi Arabia; e Mohamed Saeed Tamer Chair for Pharmaceutical Industries, King Abdulaziz University, Jeddah, Saudi Arabia; f Global Analytical and Pharmaceutical Development, Alexion Pharmaceuticals, New Haven, CT, USA; g Vaccines and Immunotherapy Unit, King Fahd Medical Research Center, King Abdulaziz University, Jeddah, Saudi Arabia; h Department of Pharmaceutics and Industrial Pharmacy, Faculty of Pharmacy, Cairo University, Cairo, Egypt; i Department of Pharmaceutics and Industrial Pharmacy, Faculty of Pharmacy, Minia University, Minia, Egypt; j Department of Pharmaceutics, Faculty of Pharmacy, Umm Al-Qura University, Makkah, Saudi Arabia; k Department of Medical Microbiology and Parasitology, Faculty of Medicine, King Abdulaziz University, Jeddah, Saudi Arabia; l Department of Pharmacology, Faculty of Pharmacy, Zagazig University, Zagazig, Egypt; m Clinical Pharmacy and Pharmacology Department, Ibn Sina National College for Medical Studies, Jeddah, Saudi Arabia; n Department of Pharmacology, Faculty of Pharmacy, Minia University, Minia, Egypt

**Keywords:** Liposomes, cancer, bee venom

## Abstract

Certain anticancer agents selectively target the nucleus of cancer cells. One such drug is 2-methoxyestradiol (2ME), which is used for treating lung cancer. To improve the therapeutic effectiveness of these agents, many new methods have been devised. 2ME was entrapped into the core of hydrophobic invasomes (INVA) covered with Phospholipon 90G and apamin (APA). The Box–Behnken statistical design was implemented to enhance the composition. Using Design-Expert software (Stat-Ease Inc., Minneapolis, MN), the INVA component quantities were optimized to obtain spherical particles with the smallest size, that is, a diameter of 167.8 nm. 2ME-INVA-APA significantly inhibited A549 cells and exhibited IC_50_ of 1.15 ± 0.04 µg/mL, which is lower than raw 2ME (IC_50_ 5.6 ± 0.2 µg/mL). Post 2ME-INVA-APA administration, a significant rise in cell death and necrosis was seen among the A549 cells compared to those treated with plain formula or 2ME alone. This effect was indicated by increased Bax expression and reduced Bcl-2 expression, as well as mitochondrial membrane potential loss. Moreover, the cell cycle analysis showed that 2ME-INVA-APA arrests the G2-M phase of the A549 cells. Additionally, it was observed that the micellar formulation of the drug increased the cell count in pre-G1, thereby exhibiting phenomenal apoptotic potential. Furthermore, it up-regulates caspase-9 and p53 and downregulates TNF-α and NF-κβ. Collectively, these findings showed that our optimized 2ME-INVA-APA could easily seep through the cell membrane and induce apoptosis in relatively low doses.

## 1. Introduction

One of the most common and rapidly increasing types of cancer is lung cancer (LC), of which non-small cell LC (NSCLC; 85%) is more common than small cell LC (SCLC; 15%) (Teramoto et al., 1999; Lemjabbar-Alaoui et al., 2015; Wei et al., 2020). One of the major causative factors of LC is smoking, while other common factors include chronic obstructive pulmonary disease (COPD), air pollution, and occupational hazards such as inhalation of toxic gases. Notably, LC is also caused, albeit rarely, due to dietary and genetic factors (Amirshahrokhi & Bohlooli, 2013). Unfortunately, LC is associated with a staggering mortality rate. Treatment plan includes surgery, adjuvant therapy, chemotherapy, and radiotherapy (Lemjabbar-Alaoui et al., 2015; Herbst et al., 2018).

**Figure 1.**
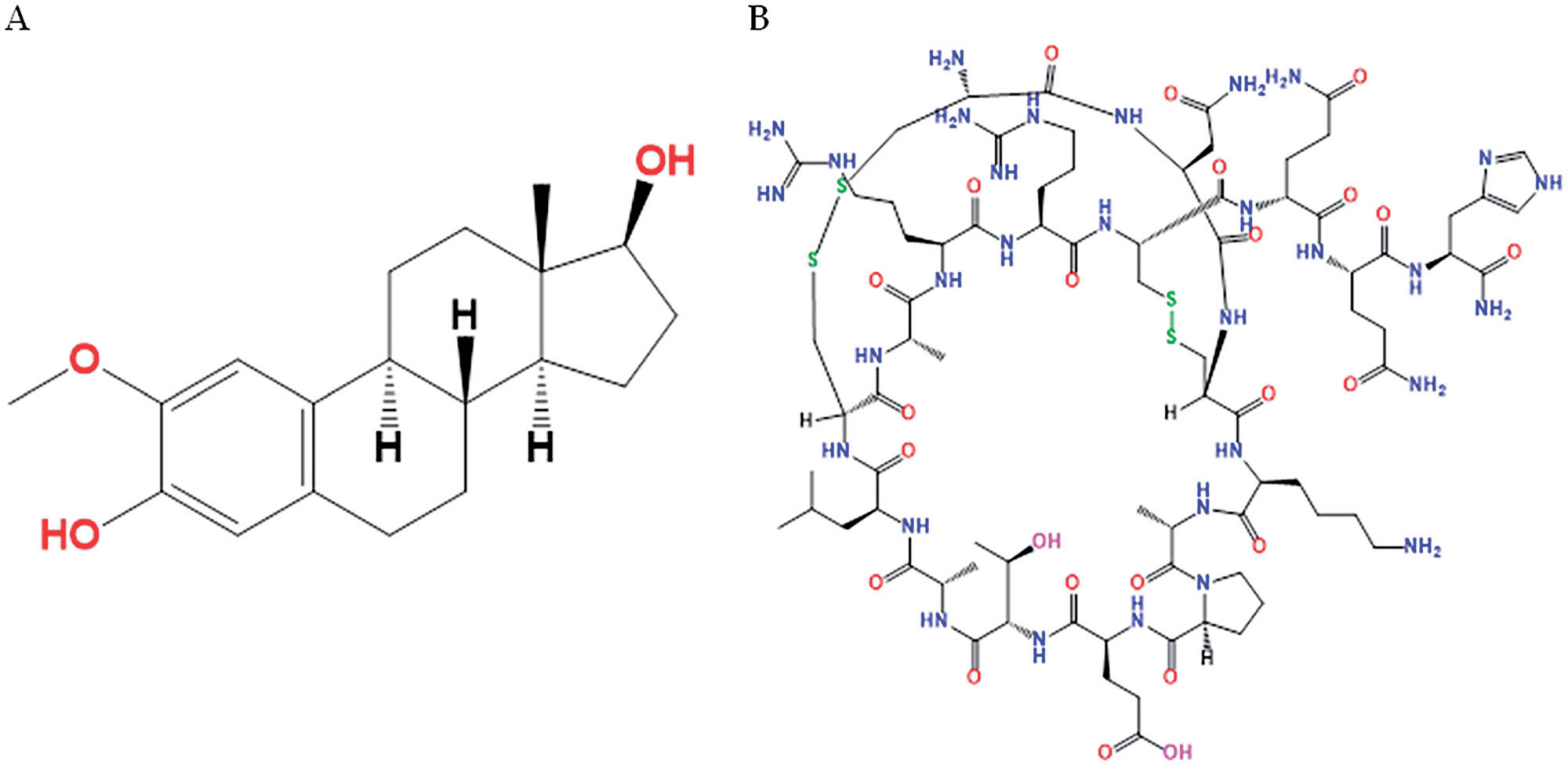
Chemical structure of 2ME (A) and APA (B).

2-Methoxyestradiol (2ME, Figure 1(A)) is a naturally occurring metabolite of estrogen, with anti-proliferative and anti-angiogenic properties, which can help arrest the growth and potentially destroy cancer cells. 2ME shows zero estrogenic efficacy (Lee et al., 2008; Tevaarwerk et al., 2009). Its anti-cancer property stems from its ability to initiate apoptosis by inhibiting hypoxia-inducible factor 1 and activating p53 (Pillai et al., 2017). Certain studies have observed that 2ME binds to tubulin, thereby stopping mitosis by forming microtubule (Lakhani et al., 2003). A report deemed 2ME effective in fighting prostate cancer, although the plasma concentration of 2ME does not sustain for long (Sweeney et al., 2005). It was noted that a significantly high dose was required in human beings when administered orally; however, nano-2ME formulation shows enhanced pharmacokinetic properties in animal test subjects (Wang et al., 2011; Alhakamy et al., 2021).

Invasomes (INVA) are innovative elastic vesicles made of several chemicals, including terpene(s), ethanol, and phosphatidylcholine (Ahmed & Badr-Eldin, 2019), which enhance the absorption of water soluble and lipid soluble drugs (Alhakamy et al., 2021). This enhancement can likely be attributed to terpenes, which break down the tightly packed lipids in epidermal (Ahmed & Badr-Eldin, 2019) and engage with intracellular proteins (Attalla et al., 1996). Similarly, ethanol helps the drugs seep through the stratum corneum. Moreover, it exhibits anti-angiogenic action through electrostatic repulsion by maintaining a negative surface charge (Ahmed & Badr-Eldin, 2019). INVA have shown promising activity against MCF-7 breast cancer cell line (Vidya & Lakshmi, 2019)

Apamin (APA, Figure 1(B)), a peptide present in bee venom (apitoxin), has been employed in the delivery of specific drugs. APA has a wide range of pharmacological activities that might be modified for therapeutic purposes (Gu et al., 2020; Alhakamy et al., 2021). Furthermore, the cytotoxic action of bee venom components has been documented.

Accordingly, the aim of this study was to formulate and optimize 2ME INVA fortified by APA that possess the least possible vesicle size. The Box–Behnken design was implemented to achieve this target. The optimized formulation was then tested for *in vitro* anticancer activity against LC cells.

## 2. Materials

For this study, 2ME was obtained from Jinlan-Pharm-Drugs Technology Co., Ltd. (Hangzhou, China); terpene (d-limonene and β-citronellol), APA, methanol, and acetonitrile from Sigma-Aldrich (St. Louis, MO); Phospholipon^®^ 90G (purified soybean lecithin with a phosphatidylcholine content of at least 90%) from Lipoid GmbH (Ludwigshafen, Germany).

## 3. Methods

### 3.1. Preparation of 2ME-INVA-APA

The preparation method built by Ahmed & Badr-Eldin (2019) was adopted to formulate 2ME-INVA-APA, but with modifications. Phospholipon^®^ 90G and 2ME (100 mg) were dissolved in a 1:2, v/v ratio of methanol/chloroform combination. The prepared solution was then rotated in a rotary evaporator (model R-200, Buchi, Essen, Germany) in order to eliminate the organic solvent. Then, a thin lipid films were formed and stored in a cabinet with vacuum system (model 5831, Thermo Fisher Scientific, Waltham, MA) overnight to remove any solvent traces. After that, terpene was applied to the performed lipid films, and then a combination of phosphate buffer saline/ethanol with a ratio of 7:3 (pH 7.4) was added. To produce a final volume of 10 mL, then the combination was rotated for 1 h at 25 °C. The resulting vesicles were kept at 25 °C for a period of time (1 h) in order to let them swell. To reach the appropriate particle size, the vesicles were ultrasonicated for 8 min per cycle for two cycles in an ice bath using a Sonics Vi-bra Cell microtip with an amplitude of 40%, 750 W, 20 kHz (Sonics & Materials Inc., Newtown, CT). Then, APA (1 mg) was applied to the vesicles, and then they were stirred for 30 min at room temperature. The obtained vesicles were stored under nitrogen.

### 3.2. Experimental design

A design software, which is Box–Behnken design with a three-factor, was used in order to adjust 2ME-INVA-APA utilizing Design-Expert version software (Version 12, Stat-Ease Inc., Minneapolis, MN). Phospholipid % (*X*_1_), ethanol % (*X*_2_), and terpene % (*X*_3_) were utilized as independent variables. Invasomes size was studied as a response (*Y*_1_). Table 1 lists each factor the coded levels, which designated as (1, 0, +1), as well as their definite values. In this experiment, 15 runs were performed. Table 2 summarizes the runs and the results. For the purpose of selecting the best fitting model for the measured response, statistical parameters such as predicted and adjusted determination coefficients, as well as an adequate precision ratio, were computed and used. The best-fitting model was represented by the corresponding polynomial equation. The measured response was statistically analyzed using analysis of variance (ANOVA) to estimate the significance of the studied variables at 95% level of significance. Perturbation and 3D plots were produced to present the main effects of the variables and the interaction between them. To reduce vesicle size, the analyzed variables were optimized using a numerical technique based on the desirability approach.

**Table 1.** Independent variables and responses utilized for the formulation of the 2ME-INVA-APA in the Box–Behnken design software.

Independent variable	Levels		
	(–1)	(0)	(+1)
*X*_1_: phospholipid %	5	10	15
*X*_2_: ethanol %	1	2	3
*X*_3_: terpene %	1	1.5	2
Response	Desirability constraints		
*Y*: vesicle size (nm)	Minimize		

2ME: 2-methoxyestradiol; INVA: invasomes; APA: apamin.

**Table 2.** Experimental runs of the prepared 2ME-INVA-APA corresponding to design Box–Behnken software and their measured vesicle size.

Run #	Independent variables			Vesicle size (nm)
	Phospholipid %	Ethanol %	Terpene %	
1	15	2	2	368.7
2	10	2	1.5	279.4
3	10	2	1.5	268.3
4	5	1	1.5	229.3
5	15	1	1.5	418.3
6	15	3	1.5	296.5
7	5	2	1	191.7
8	5	3	1.5	173.2
9	10	3	2	251.8
10	10	1	1	289.7
11	10	3	1	219.9
12	15	2	1	311.4
13	10	1	2	328.1
14	5	2	2	213.1
15	10	2	1.5	272.1

2ME: 2-methoxyestradiol; INVA: invasomes; APA: apamin.

### 3.3. Vesicle size measurement

The vesicular size and polydispersity index (PDI) of 2ME-INVA-APA (Zetasizer Nano ZSP, Malvern Panalytical, Malvern, UK) were measured using the dynamic light scattering technique and calculated as the mean of five determinations. The laser wavelength was 633 nm, scattering angle of 173, temperature of 25 °C, medium viscosity of 0.8872 cP, and medium refractive index of 1.33.

### 3.4. Calculating IC_50_ through MTT assay

The MTT viability assay [3-(4,5-dimethylthiazol-2-yl)-2,5-diphenyltetrazolium bromide] was conducted in order to obtain the IC_50_ values of untreated A549 cells (control) after treatment with blank-INVA (INVA-APA), 2ME, or 2ME-INVA-APA for 48 h (Caruso et al., 2017). Then, in a 96-well plate, A549 cells (1 × 10^5^ cells) were seeded and incubated overnight. Afterwards, these were treated with the plain (INVA-APA), 2ME, or 2ME-INVA-APA at (0.39 μM, 1.56 μM, 6.26 μM, 25 μM, and 100 μM). After 48 hours, the utilized medium was changed to solution (MTT) with the concentration of 2 mg/mL, and then prepared plates were placed in an incubator for 4 h at 37 °C. After that, 200 µL of 100% DMSO was added in order to eliminate the formazan, and then the plates were incubated in a 5% CO_2_ at 37 °C for 5 min. Then, a microplate reader (Spark^®^ multimode, Tecan Group Ltd., Maennedorf, Switzerland) identified an absorbance at 569 nm in each well. The results were presented in terms of percent cell viability relative to the control. Following the plotting of dose response curves, GraphPad Prism (GraphPad, Inc., La Jolla, CA), was used to define the IC_50_ value for each of the experimental condition.

### 3.5. Cell cycle analysis

The cell cycle phases of A549 cells were studied using flow cytometry (FACScalibur, BD Bioscience, Franklin Lakes, NJ) (Alfaifi et al., 2020). Six-well plates were utilized to seed the cells in a density of 3 × 10^5^ cells/well. These were divided into two: untreated (control) or treated group (treated with plain (INVA-APA), 2ME, or 2ME-INVA-APA) left for 24 hours. Next, the cells were washed; 70% cold ethanol was used to fix the cells, and RNase staining buffer and propidium iodide (PI) were used to stain them. Flow cytometry resulted in 10,000 gated events. MultiCycle AV was used to analyze the data (Phoenix Flow Systems, San Diego, CA).

### 3.6. Annexin V-FITC apoptosis assay

In this part, an Annexin V-FITC Apoptosis Detection kit (BioVision, Cambridge BioSciences, Cambridge, UK) was utilized in order to observe the apoptotic effects of INVA-APA, 2ME, and 2ME-INVA-APA on A549 cells. Six-well plates were used to seed the cells at a density of 1 × 10^6^ cells/well. These were split into two: untreated (control) and treated group (treated with plain, INVA-APA, 2ME, or 2ME-INVA-APA) left for 24 hours. Following this, the cells were first centrifuged to separate them. Then, PBS buffer was used to wash them. After washing, 500 μL of 1× binding buffer was applied to the cells. Then, they were stained with PI and annexin V-FITC for flow cytometry. For each treatment, flow cytometry yielded a minimum of 20,000 events. The data were analyzed using MultiCycle AV.

### 3.7. Real-time polymerase chain reaction (RT-qPCR)

#### 3.7.1. RNA extraction

A Qiagen RNeasy mini kit (Qiagen, Manchester, UK) RNA was utilized to extract the RNA from the selected cells (A594 cells). The RNA’s purity and concentration were verified using a Nanodrop Spectrophotometer (ND-2000C, Thermo Fisher Scientific, Waltham, MA). All RNA samples had an *A*_260 nm_/*A*_230 nm_ ratio of not less than 1.8 and an *A*_260 nm_/*A*_280 nm_ ratio of not less than 1.9.

#### 3.7.2. CDNA synthesis and PCR amplification

A Green kit (iScript™ One-Step RT-PCR Kit With SYBR^®^ (BioRad, Hercules, CA)) was utilized (cDNA) in order to normalize RNA between tubes and reverse transcribe it to complementary DNA. The expression patterns of p53, Bax, Bcl-2, casp3, TNF- and NFB, and -actin were studied using 10 ng of RNA template in a 50-mL reaction mixture of iScript one-step RT-PCR kit with SYBR^®^ Green mix and a 7500 Fast real-time PCR machine (Applied Biosystems, Thermo Fisher Scientific, Waltham, MA).

#### 3.7.3. Mitochondrial membrane potential (MMP)

The MMP levels in A549 cells seeded in 96-well plates (1 × 10^5^ cells/well) and treated to INVA-APA, 2ME, or 2ME-INVA-APA for 24 hours were monitored using an assay kit (MitoProbe™ TMRM) (Alhakamy et al., 2020). INVA-APA, 2ME, or 2ME-INVA-APA were used at IC_50_ concentrations.

### 3.8. Statistical analysis

GraphPad Prism (GraphPad, Inc., La Jolla, CA) was used to conduct the statistical analysis. The data were given as mean standard deviation. To compare the means, an ANOVA was used, followed by Tukey’s post hoc test. Statistical significance was defined as *p*=.05.

## 4. Results and discussion

### 4.1. Experimental design

**Figure 2.**
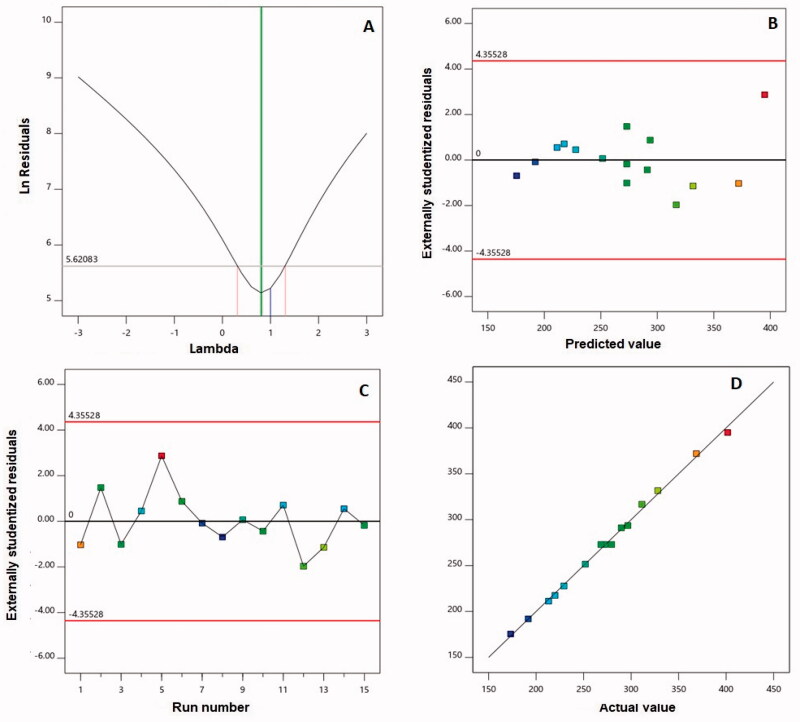
Diagnostic plots for particle size of 2ME-INVA-APA. (A) Power transforms Box–Cox plot for, (B) outwardly predicted vs. studentized residuals values, (C) outwardly run number vs. studentized residuals, and (D) actual values plot vs. predicted. 2ME: 2-methoxyestradiol; INVA: invasomes; APA: apamin.

The Box–Behnken is a reliable design used in this work to prepare and optimize 2ME-INVA-APA to obtain the smallest vesicle size. Due to its highest determination coefficient *R*^2^, the two-factor interaction model was the best fit for the vesicle size data. Furthermore, the predicted and adjusted *R*^2^ values were in high agreement, indicating that the model was valid. The signal-to-noise ratio was measured using adequate precision, and the computed ratio of 66.71 suggests that the chosen model can be utilized to follow the design space. Figure 2 illustrates the diagnostic plots produced to assess the goodness of 2FI interaction model fit. Figure 2(A) shows a value of the recommended lambda (*λ*) (0.81) in the Box–Cox plot for power transforms. The present value of 1 is contained in the 95% confidence interval (indicated by the red bounds); no specific transformation for measured size is suggested (Singh et al., 2011; Badr-Eldin et al., 2021). The calculated maximum to minimum size ratio of 2.31 supports the absence of transformation, where a ratio more than 10 is likely to need transformation. Within the bounds of the externally studentized residuals vs. predicted response plot, as shown in Figure 2(B), a random distribution of the measured globule size suggests that no continuous error exists. Furthermore, the residual vs. run plot, as shown in Figure 2(C), shows the distribution of random points, showing that no lurking variable may affect the measured size. The observed vs. predicted values of vesicle size, as shown in Figure 2(D), are in good agreement. This further validates the chosen design model and its potential use in forecasting the optimal formula that meets the specified requirement (Alhakamy et al., 2020).

#### 4.1.1. Influence of variables on size

The prepared 2ME-INVA-APA exhibited vesicular size ranging from 173.2 to 418.3 nm. The equation of the polynomial (Equation (1)) representing the 2FI sequential model in terms of coded factors was generated.

(1) $$Y=272.99+71.36X_{1}–38.41X_{2}+18.63X_{3}–12.25X_{1}X_{2}+8.97X_{1}X_{3}–1.63X_{2}X_{3}$$

**Figure 3.**
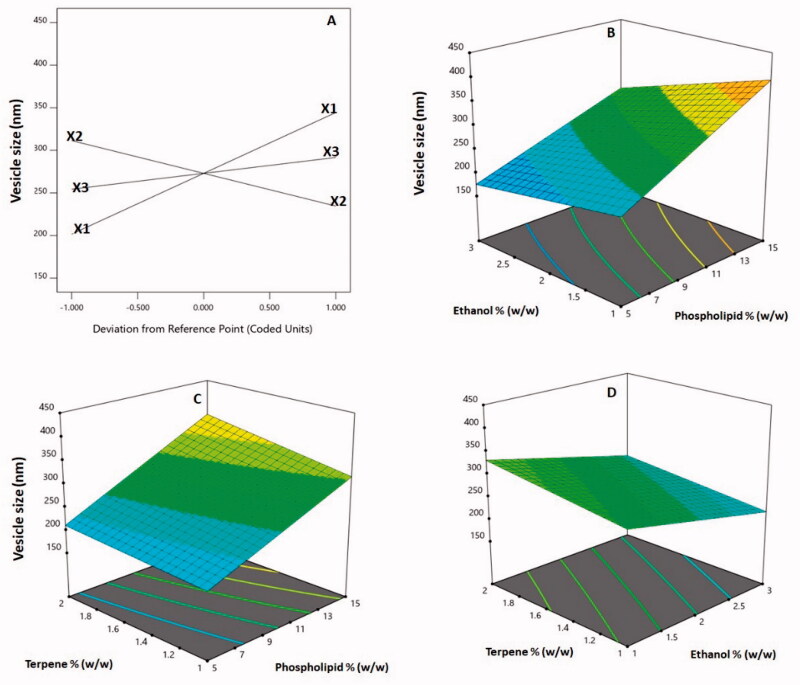
Perturbation plot (A) and three-dimensional surface plots (B–D) for the effect and interactions between phospholipid % (*X*_1_), ethanol % (*X*_2_), and terpene % (*X*_3_) on vesicle size of 2ME-INVA-APA. 2ME: 2-methoxyestradiol; INVA: invasomes; APA: apamin.

Table 3 shows that the linear factors *X*_1_ (phospholipid %), *X*_2_ (ethanol %), and *X*_3_ (terpene %) had a significant influence on vesicle size at *p*<.05. Furthermore, at the same level of significance, the interaction variables *X*_1_*X*_2_ and *X*_1_*X*_3_, which describe the interaction between phospholipid percentage and either ethanol or terpene percentage, were significant. The perturbation plot, presented in Figure 3(A), shows the independent variables effects on the vesicle size. Both variables *X*_1_ and *X*_3_ had a positive slope, showing a direct link between the variable and the response. The variable *X*_2_ had a negative slope, indicating that ethanol % is inversely related to size. The positive sign of the linear components *X*_1_ and *X*_3_ and the negative sign of the linear term *X*_2_ confirm the observed trend. Figure 3(B–D) shows 3D response surface plots for the tested factors' influence on vesicle size, revealing that phospholipid percentage had the most significant effect on vesicle size. This is supported by the greatest coefficient of the term *X*_1_ in the constructed equation.

**Table 3.** Measured vesicle size statistical analysis output of 2ME-INVA-APA according to two factor interaction model.

*R* ^2^	Adjusted *R*^2^	Predicted *R*^2^	Adequate precision	Sequential *p* value	Lack of fit *p* value
0.9884	0.9943	0.9872	21.1491	0.0017	0.7169
*p* Value of significant terms	*X*_1_	*X*_2_	*X*_3_	*X*_1_ *X*_2_	*X*_1_ *X*_3_
	<.0001	<.0001	<.0001	.0009	.0058

2ME: 2-methoxyestradiol; INVA: invasomes; APA: apamin.

### 4.2. Optimization of 2ME-INVA-APA

The optimum formulation composition was predicted using Design Expert software^®^ (Stat-Ease Inc., Minneapolis, MN) through numerical optimization with the purpose of decreasing particle size. Table 4 shows the composition of optimized 2ME-INVA-APA. With a percent error of 2.15%, the measured vesicle size was in good agreement with the predicted value, demonstrating the design's suitability for the optimization process.

**Table 4.** The optimized 2ME-INVA-composition, APA’s as well as its expected and actual vesicle size.

Variables	*X*_1_: phospholipid %	*X*_2_: ethanol %	*X*_3_: terpene %
Optimum values	5.32	2.95	1.06
Vesicle size (nm)	Predicted value	Observed value	Error %
	171.5	167.8	2.15

### 4.3. Cytotoxicity assay

**Figure 4.**
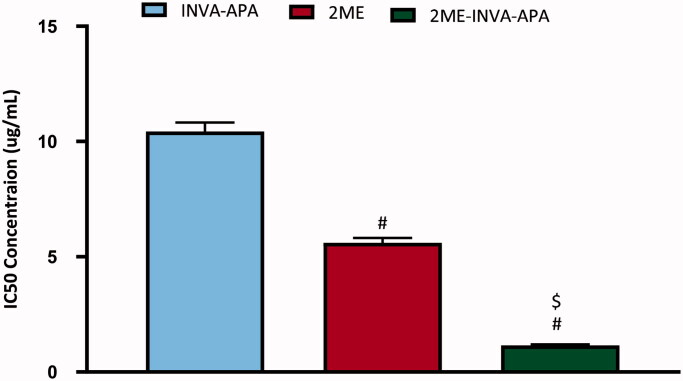
IC_50_ of the INVA-APA, 2ME, and 2ME-INVA-APA in the A549 cells. The results are the average of four separate trials with standard errors. ^#^Significantly different from INVA-APA (*p*=.05). ^$^Compared to 2ME, there is a significant difference (*p*=.05).

MTT assay revealed that INVA-APA, 2ME, or 2ME-INVA-APA inhibited A549 cells in a dose-dependent manner. INV-APA and 2ME demonstrated inhibition of the viability of the cells with IC_50_ values of 10.43 ± 0.39 and 5.6 ± 0.2 µg/mL, respectively (Figure 4). 2ME-INVA-APA significantly inhibited A549 cells as compared to the other two groups (*p* < .05) and exhibited an IC_50_ value of 1.15 ± 0.04 µg/mL.

### 4.4. Cell cycle analysis

**Figure 5.**
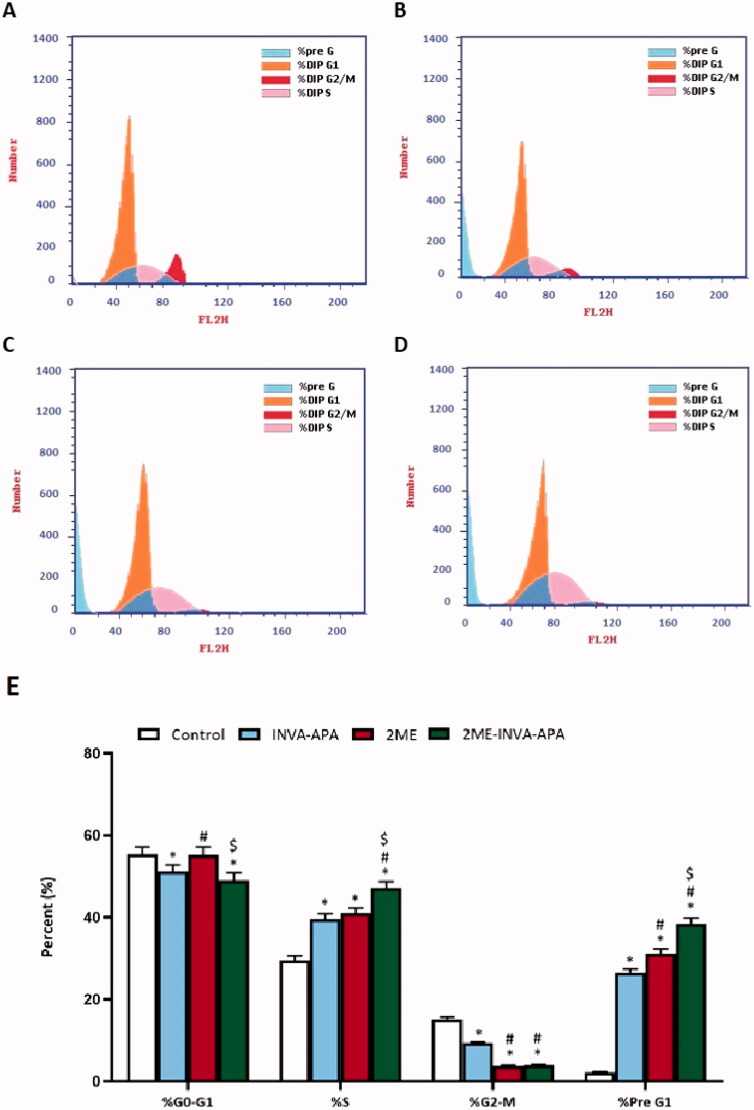
Graphical presentation of A549 cell cycle phases following treatment for 24 h using flow cytometry analysis. (A) control; (B) INVA-APA; (C) 2ME; (D) 2ME-INVA-APA; (E) graphical presentation of cell cycle phases. All data is given as the mean standard deviation of three separate studies. *p=.05 was considered significant when compared to the control. ^#^*p*<.05 was considered significant when compared to INVA-APA and ^$^*p*<.05 was considered significant when compared to 2ME.

Cell cycle analysis test was conducted in order to verify whether 2ME-INVA-APA inhibited A549 cells by influencing the cell cycle phases. The A549 cells (control) demonstrated proliferative profile with around 55% at G0/G1 phase, 2% at pre-G1 phase, 15% at G2 phase, and 30% at S phase (Figure 5(A)). INVA-APA and 2ME treatments significantly enhanced the number of cells in the pre-G1 and S phases of the cell cycle (*p* < .05) (Figure 5(B,C)). The percentage of cells at G2/M decreased to 9.29 ± 0.28% and 3.75 ± 0.16% for INVA-APA and 2ME treatments, respectively, indicating that they arrested the cell cycle at S phase. Interestingly, 2ME-INVA-APA significantly enhanced the S (47.15 ± 1.54%) and pre-G1 (38.42 ± 1.45%) phases, indicating significant arrest of A549 S phase when compared with other groups (Figure 5(D,E)).

### 4.5. Annexin V apoptosis assay

**Figure 6.**
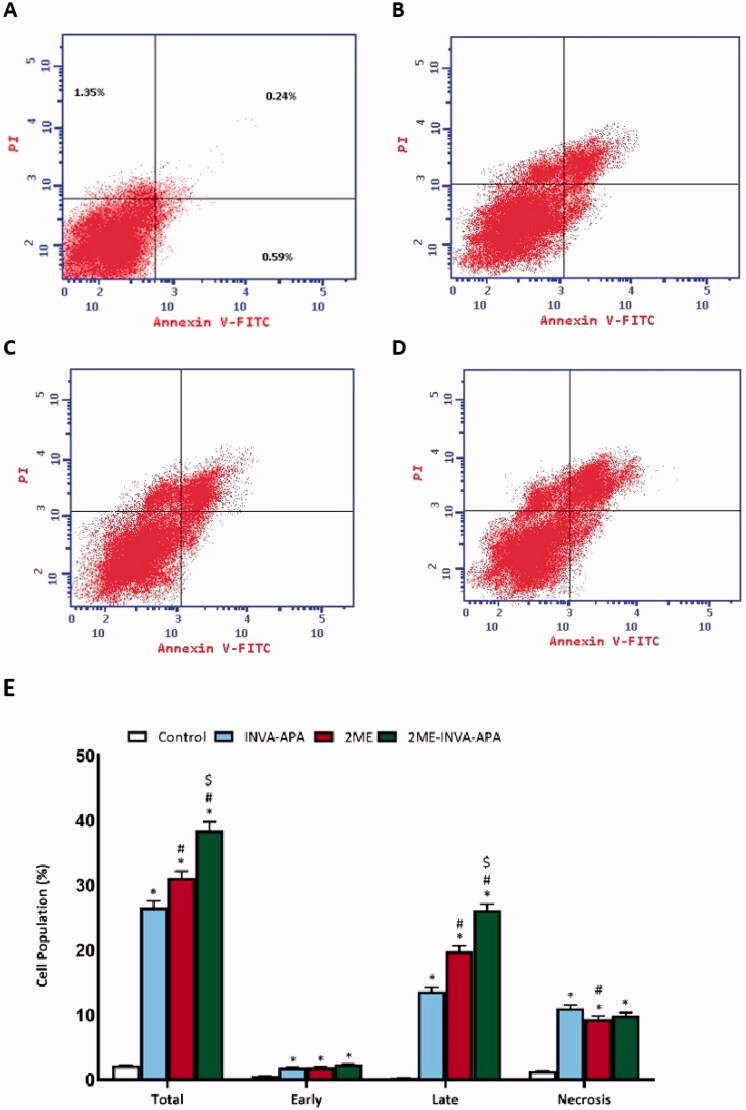
Analysis of apoptosis via Annexin-V-FITC staining in A549 cells. The data are stated as the mean ± SE of number of separate tests (three). (A) control; (B) INVA-APA; (C) 2ME; (D) 2ME-INVA-APA; (E) graphical presentation of cell populations. *p< .05 considered significantly different from the control. ^#^*p*< .05 considered significantly different from INVA-APA and ^$^*p*< .05 considered significantly different from 2ME.

Annexin V and PI staining were used to assess the apoptotic rate (Figure 6(A–E)). 2ME-INVA-APA significantly increased the percentage of early/late apoptosis and necrosis (*p* < .05) (Figure 6) compared to 2ME.

### 4.6. INVA-APA modulating Bax, Bcl-2, caspase 3, and p53 protein levels

**Figure 7.**
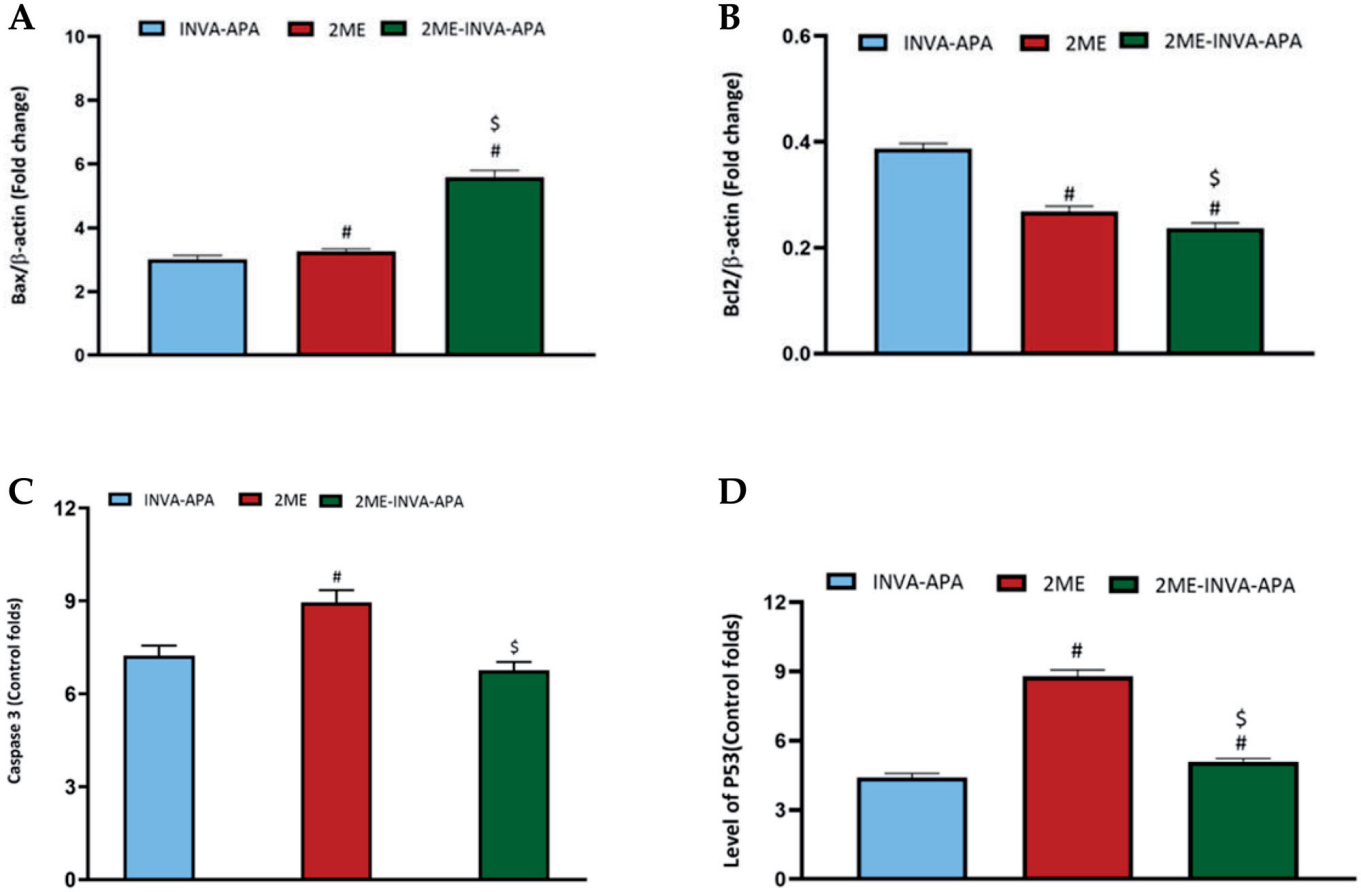
Expression of apoptotic markers (A) Bax, (B) Bcl-2 (C) caspase-3, and (D) p53 within A549 lung cancer cells. All results are stated as the mean ± SE of three separate tests. ^#^*p*<.05 was considered significant when compared to INVA-APA and ^$^*p*<.05 was considered significant when compared to 2ME.

A549 cells treated with the optimized formula revealed a significant (*p*<.05) increase in the expression of Bax and p53 (Figure 7(A,D)). Instead, it decreased the expression of the antiapoptotic protein Bcl-2 significantly compared to plain INVA APA (Figure 7(B)). However, no significant effect was observed on caspase 3 expression in the cells treated with the optimized formula compared to plain formula (Figure 7(C)). Meanwhile, 2ME alone significantly enhanced the caspase 3 expression. The effect of the optimized formula was more prominent on Bax and Bcl2 compared to 2ME, while 2ME had more prominent influence on caspase 3 and p53 than the optimized formula.

### 4.7. Expression of TNF-α and NF-κB

**Figure 8.**
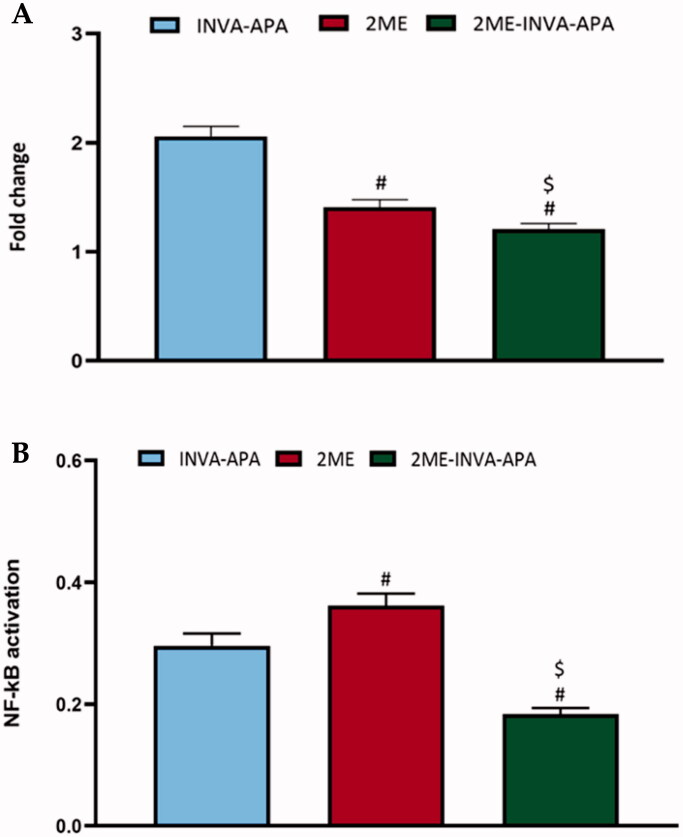
Modulation of treatments using the plain formula, 2ME-raw and 2ME-INVA-APA over expression level of inflammatory markers (A) TNF-α and (B) NF-κB. All results are expressed as the mean ± SE of three separate experiments. *Significantly different from the control (*p*<.05). ^#^*p*=.05 was considered significant when compared to INVA-APA, and ^$^*p*<.05 was considered significant when compared to 2ME.

As shown in Figure 8, treatment of A549 cell line with 2ME-INVA-APA resulted in a significant (*p*<.05) decrease in the expression of TNF-α (Figure 8(A)) and a significant (*p*<.05) reduction in the expression of activated NF-κB (Figure 8(B)) when compared with plain formula and 2ME.

### 4.8. Mitochondrial membrane potential study

**Figure 9.**
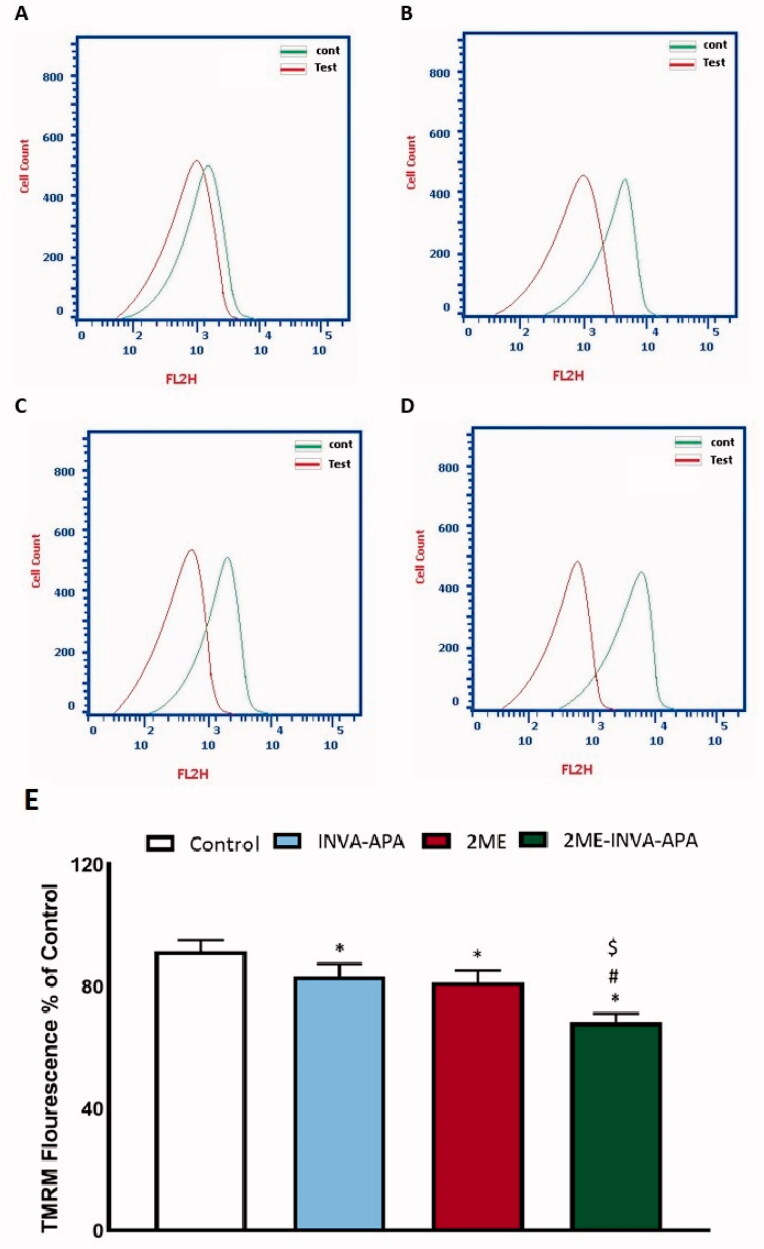
The effect of INVA-APA, 2ME-raw, and 2ME-INVA-APA on MMP variation in A549 cell. Normalization of values took place with respect to untreated (control) A549 cells and expressed as variation percentage. Data denote the mean of four independent experiments ± SE. *Significantly different vs. control (*p*<.05), ^#^significantly different vs. plain formula (*p*<.05), and ^$^*p*<.05 considered significantly different from 2ME.

To investigate the pro-apoptotic and anti-proliferative activity of 2ME-INVA-APA and its effect on MMP, the percentage variation with respect to MMP caused by varying treatments was analyzed. 2ME-INVA-APA resulted in significant changes in MMP as compared to control cells and cells treated with the plain formula (Figure 9(A–E)).

## 5. Discussion

2ME is an anti-angiogenic, anti-proliferative, and pro-apoptotic medication with a lot of promise (Fotsis et al., 1994; Becker et al., 2008). As it suppresses the proliferation of many human cancer cell lines *in vitro*, it might be useful in the treatment of cancer (Benedikt et al., 2010; Bu et al., 2022); however, it was found to exhibit very poor oral bioavailability and extensive metabolism (Potter, 2018). Our study was conducted to improve the therapeutic effectiveness of 2ME through its inclusion into the core of hydrophobic INVA covered with Phospholipon 90G and APA to produce 2ME-INVA-APA.

The Box–Behnken statistical design was implemented to enhance and optimize the composition. The Box–Behnken is a rotatable or nearly-rotatable three-level response surface design, commonly used in pharmaceutical research for optimization (Khuri & Mukhopadhyay, 2010). The observed increase in vesicle size with increasing phospholipid percentage is in good agreement with previous studies for vesicular formulations. PL percent had a favorable influence on the vesicle size of ethosomes loaded with anti-psoriatic drug, according to Dubey et al. (2007). Ahmed & Badr-Eldin (2019) found that raising Phospholipon concentration increased the vesicle size of avanafil-INVA. Furthermore, an increase in size at higher terpene percentages has been previously reported for INVA (Dragicevic-Curic et al., 2008; Nangare & Dugam, 2020).

After optimization of the formula, the effectiveness of 2ME-INVA-APA was investigated on A549 cells and compared with 2ME and plain formula. Our results showed that 2ME-INVA-APA inhibited A549 cells and its IC_50_ value was significantly lower than IC_50_ values of 2ME and plain formula. Therefore, we can speculate that 2ME-INVA-APA could easily seep through the cell membrane and induce apoptosis despite a low dose.

Regarding cell cycle analysis, we observed that 2ME arrested the G2-M phase of A549 cells, which is in agreement with findings reported by Lee et al. (2008). Nevertheless, a further enhanced effect was seen using the optimized formula. In addition, the optimized formula resulted in a noticeable increase in the number of cells in the pre-G1 phase, which is also in agreement with previous literature (Lee et al., 2008). This increase is a characteristic sign of apoptotic potential (Mascitelli & Pezzetta, 2007; Hardie et al., 2016; Rady et al., 2017; El-Aarag et al., 2019). Notably, blank-INAVA APA decreased the cell count in the S-phase.

The apoptotic effect of the test compounds was confirmed by Annexin V staining and MMP assay. 2ME-INVA-APA significantly increased the percentage of early/late apoptosis and necrosis and resulted in significant changes in MMP. These changes in A549 profile indicate apoptosis by 2ME-INVA-APA. Wei et al. reported similar apoptotic effect for 2ME at 10 µg/mL when A549 cells were treated for 24 h. This effect has also been reported in human LC cell line H1299; however, the effect was reduced at 48 µg/mL (Wei et al., 2020).

In order to investigate the pathways that may be involved in the pro-apoptotic effect of 2ME-INVA-APA, we assessed the relative expression of Bax, Bcl-2, caspase 3, and p53. Significant upregulation of Bax and downregulation of Bcl-2 was observed in the A549 LC cells treated by 2ME-INVA-APA. This could be attributed to the increased apoptosis in the 2ME-INVA-APA treated cells. Previous studies has reported similar effect of 2ME on Bax and Bcl-2 expression (Badr-Eldin et al., 2020; Alhakamy et al., 2021). These findings substantiate 2ME-INVA-APA’s anti-neoplastic ability. P53 is a tumor suppressor gene that increases when it senses potentially oncogenic stimuli. When p53 mutates, its activity is decreased, leading to uncontrolled cell division (Li & Yuan, 2008). The optimized formula increased the expression of p53 in A549 cells, indicating that p53 plays a critical role in 2ME-INVA-mediated apoptosis at very low dose in A549 cell line.

Caspases are a broad family of cysteine proteases that are required for intrinsic apoptosis initiation and execution. A frequent downstream apoptosis effector is caspase-3 (Farnebo et al., 2010). Caspase-3 expression was upregulated in 2ME treated cells, while the effect of the optimized formula was comparable to the plain formula. This may be due to the low concentration of 2ME in the optimized formula. A previous study showed that at least 10 μM of 2ME was required to increase caspase-3 and the increase was gradual as well. This demonstrates that the intrinsic-mediated caspase-3 activation pathway is involved in 2ME mediated apoptosis (Tang et al., 2020). We reported that 2ME-INVA-APA downregulated the expression of both TNF-α and NF-κB. This effect highlights the anti-angiogenic and anti-inflammatory effects of 2ME. Moreover, the inhibition of TNF-α and upregulation of p53 by the optimized formula could make a breakthrough in cancer management (Mascitelli & Pezzetta, 2007; El-Aarag et al., 2019). TNF-α is a pleiotropic proinflammatory cytokine that regulates a variety of physiological and pathological signaling pathways including as inflammation, differentiation, proliferation, and apoptosis induction. TNF-α promotes cancer cell proliferation, migration, and adhesion in the tumor microenvironment, accelerating multistep cancer development (Liu et al., 2019).

The enhanced anti-neoplastic property of 2ME-INVA-APA can be attributed to increased release of 2ME from the INVA, as well as increased seepage through cell membrane due to the small size (Ali et al., 2013; Hua et al., 2016). Furthermore, similar potential of APA and 2ME against A549 LC cells have been reported in literature (Wang et al., 2011; Fahmy, 2018). Similarly, INVA delivery of drug-free formulation in A549 cells induces rapid necrosis.

## 6. Conclusions

The Box–Behnken design was implemented successfully to optimize 2ME-INVA-APA formulation with minimized size. The particle size increased with increasing both phospholipid and terpene concentrations, while it decreases at higher ethanol percentages. The optimized formulation with size of 167.8 nm showed enhanced cytotoxicity and apoptosis against A549 cells. Further, it significantly suppressed inflammatory marker activity. Therefore, the proposed formulation is a promising anti-neoplastic agent for the treatment of LC.
